# Multipeptide stimulated PBMCs generate T_EM_/T_CM_ for adoptive cell therapy in multiple myeloma

**DOI:** 10.18632/oncotarget.28067

**Published:** 2021-09-28

**Authors:** Trupti Vardam-Kaur, Latha B. Pathangey, Daniel J. McCormick, P. Leif Bergsagel, Peter A. Cohen, Sandra J. Gendler

**Affiliations:** ^1^Department of Immunology, Mayo Clinic, Scottsdale, AZ, USA; ^2^Department of Biochemistry and Molecular Biology, Mayo Clinic, Scottsdale, AZ/Rochester, MN, USA; ^3^Department of Hematology and Oncology, Mayo Clinic, Scottsdale, AZ, USA; ^*^Deceased

**Keywords:** multiple myeloma, cellular therapy, CD4/CD8 T cells, peptides, immunotherapy

## Abstract

Multiple Myeloma (MM) patients suffer disease relapse due to the development of therapeutic resistance. Increasing evidence suggests that immunotherapeutic strategies can provide durable responses. Here we evaluate the possibility of adoptive cell transfer (ACT) by generating *ex vivo* T cells from peripheral blood mononuclear cells (PBMCs) isolated from MM patients by employing our previously devised protocols. We designed peptides from antigens (Ags) including cancer testis antigens (CTAs) that are over expressed in MM. We exposed PBMCs from different healthy donors (HDs) to single peptides. We observed reproducible Ag-specific cluster of differentiation 4^+^ (CD4^+^) and CD8^+^ T cell responses on exposure of PBMCs to different single peptide sequences. These peptide sequences were used to compile four different peptide cocktails. Naïve T cells from PBMCs from MM patients or HDs recognized the cognate Ag in all four peptide cocktails, leading to generation of multiclonal Ag-specific CD4^+^ and CD8^+^ effector and central memory T (T_EM_ and T_CM_, respectively) cells which produced interferon-gamma (IFN-γ), granzyme B and perforin on secondary restimulation. Furthermore, this study demonstrated that immune cells from MM patients are capable of switching metabolic programs to induce effector and memory responses. Multiple peptides and cocktails were identified that induce IFN-γ^+^, T1-type, metabolically active T cells, thereby paving the way for feasibility testing of ACT in phase I clinical trials.

## INTRODUCTION

Multiple Myeloma (MM) is a plasma cell malignancy mostly affecting elderly people. It is characterized by clonal proliferation of terminally differentiated antibody-producing plasma cells in the bone marrow, leading to osteolytic bone lesions. It is the second most common malignancy among hematological cancers with an incidence rate of 4.5–6 per 100,000 individuals per year [1]. The global rate of incidence and death has increased by 126% and 94%, respectively, from 1990–2016 [2]. Despite the availability of various therapeutic regimens, MM remains an incurable disease and patients succumb to it mainly due to development of resistance attributed to the constantly evolving genomic landscape in the plasma cells/tumor microenvironment and the cross-talk between them [3, 4]. The genetic instability correlates with the somatic mutation load, which in turn is proportional to the neoantigen load that is associated with a shorter progression free survival following induction therapy in MM patients [5]. This scenario suggests defective T cell recruitment and activation, thereby necessitating the development of alternative therapies that exploit the potential of neoantigens.

Novel immunotherapies comprised of chimeric antigen receptors modified-T (CAR-T) cells have given encouraging results, especially in the treatment of hematological cancers. In a recently conducted clinical trial of relapsed and refractory MM patients treated with biepitope-targeting CAR-T cells, an overall response rate of 88.2% with clinically manageable adverse effects was observed. However, CAR-T cells have limitations – only surface antigens (Ags) are targeted, poor cell persistence, exhaustion of CAR-T cells, loss of target Ags and manufacturing difficulties [6, 7]. These limitations, in addition to defective recruitment and activation, can potentially be overcome by adoptively transferring activated T cells exposed to peptides designed from multiple Ags [8]. We hypothesize that adoptive cell transfer (ACT) of large numbers of optimally *ex vivo* activated T cells will be effective in eradicating MM, bypassing the immune editing observed with vaccination therapies. We targeted cancer testis antigens (CTAs) in addition to Ags that are selectively expressed in plasma cells. Studies suggest that myeloma patients frequently express CTAs and their expression is further enhanced during relapse [9]. CTAs are generally not expressed in normal tissue except in gametes and trophoblasts, which may reduce off-tumor toxicity [10]. Furthermore, most of the CTAs support tumor growth and malignant phenotype, which reduces the likelihood of these Ags being downregulated. We therefore focused on CTAs in addition to Ags that have plasma cell restricted expression.

Peptides were designed from numerous Ags based on their level of expression and role in MM pathogenesis: B Cell Maturation Antigen (BCMA), Mucin1 (MUC1), Fc Receptor Like 5 (FcRH5), Myeloid Cell Leukemia 1 (MCL1), Receptor for Hyaluronan-Mediated Mobility (RHAMM), Self-Ligand Receptor of the Signaling Lymphocytic Activation Molecule Family 7 (SLAMF7), spliced isoform of X-Box Binding Protein 1 (XBP(S)1), Cancer Testis Antigen (CT45), Melanoma Antigen Family 3/6 (MAGEA3/6), New York Esophageal Squamous Cell Carcinoma 1 (NY-ESO-1), SEPTIN9 (SEPT9) and Wilms Tumor 1 (WT1). The significantly enhanced expression of BCMA on MM has been shown to be crucial for its growth and survival which makes it an attractive target [11]. It has already been clinically validated with the approval of anti-BCMA antibody (belantamab mafodotin) and CAR-T therapy (idecabtagene vicleucel). MUC1, initially thought to be an epithelial protein, is expressed in hematopoietic cells [12]. Aberrant expression of MUC1 mediates MYC oncogenic activation, and immunosuppression, thereby playing a vital role in progression and survival of MM patients [13, 14]. Hence, this study examined the efficacy of previously designed peptides for MUC1 (sperm protein, enterokinase and agrin domain - SEA1, SEA2 and SEA3) in inducing T cell-mediated immunity [15]. In MM, the expression of RHAMM is elevated and is associated with cytogenetic abnormalities, aggressive disease, and reduced survival [16]. FcRH5 expression is restricted to mature B cells and is particularly elevated in MM cells. Antibodies targeting FcRH5 are already in clinical development [17]. Overexpression of SLAMF7 correlates with uncontrolled proliferation of MM and antibody targeting SLAMF7 (elotuzumab) has been approved for the treatment of MM [18]. IL-6-mediated upregulation of anti-apoptotic protein, MCL1, leads to relapse [19]. MCL1 inhibitors are showing exciting pre-clinical activity in MM, and are entering clinical trials [20]. Genetic and correlative human data suggest a role for XBP(S)1 in the development of monoclonal gammopathy of unknown significance (MGUS) and MM [21]. XBP(S)1 is a downstream target of IL-6 involved in the differentiation of normal B cells into plasmablastic cells [22]. Its expression is upregulated in MM patients. CT45 expression is associated with a worst prognosis in MM [23]. It promotes malignancy by regulating cell morphology, adherence, and migration and could be a potential immunotherapeutic target [23, 24]. MAGEA3 promotes MM growth by preventing apoptosis [25]. NY-ESO-1, an immunogenic CTA, induces spontaneous immune responses, and its expression correlates with poor prognosis in MM [26]. SEPT9, a gene that encodes SEPTIN9, is hypermethylated in colorectal cancer and is being proposed for non-invasive screening purposes [27]. Preliminary evidence suggests that SEPT9 is downregulated in MM [28]. The increase in WT1 transcripts correlates with the worsening of clinical factors and stage, and Azuma et al. have shown that MM cells are highly sensitive to the granule exocytosis pathway mediated by WT1-specific cytotoxic T lymphocytes [29].

Our goal was to design peptides from MM Ags including CTAs to generate cluster of differentiation 4^+^ (CD4^+^) and CD8^+^ T cell responses from peripheral blood mononuclear cells (PBMCs), a widely available source of T cells. The data obtained served as a guideline to design multiple peptide cocktails compiled from different Ags to optimally activate T cells from unfractionated PBMCs from MM patients and healthy donors (HDs). Further, we evaluated the phenotypic, functional and metabolic characteristics of the CD4^+^ and CD8^+^ T cell populations after *ex vivo* expansion. We first designed peptides spanning 17–41 amino acids in length using NetMHCpan, a high-throughput computational platform that draws information from the Immune Epitope Database to predict peptide-major histocompatibility complex I/II (MHC I/II) binding interactions for activating both CD4^+^ and CD8^+^ T cells [15]. We employed our recently developed MHC-independent, two-step culture protocol to generate large numbers of tumor Ag-specific natural and unedited T cells from patient PBMCs [15]. We observed that many peptides induced an Ag-specific CD4^+^ and CD8^+^ T cell response leading to development of effector memory (T_EM_) and central memory (T_CM_) T cells. Based on the efficacy of peptides to stimulate T cells in HD PBMCs, we developed multiple peptide cocktails (CT) for stimulating PBMCs from MM patients. The different peptide cocktails induced T cell responses consistently in PBMCs from HDs as well as MM patients. Furthermore, the CD4^+^ and CD8^+^ T cells harvested at the end of the culture period exhibited similar extracellular acidification rates (ECAR) and oxygen consumption rates (OCR), suggesting that the disease status does not affect the metabolic machinery *ex vivo*.

## RESULTS

### Synthetic peptides designed using NetMHCpan server

Peptides were designed from the following Ags: BCMA, MUC1, FcRH5, MCL1, RHAMM, SLAMF7, XBP(S)1, CT45, MAGEA3/6, NY-ESO-1, SEPT9 and WT1. NetMHCpan server employs artificial neural network to predict the binding affinity of peptides to MHC I or II [15]. In Supplementary Figure 1A, the regions highlighted in blue represent MHC I hotspots, whereas those in red indicate high binding affinity to MHC II. The peptide length ranged between 17–41 mers and included high binding affinity for both MHC I and II. These peptides covered class I and II alleles from 90% of the US population encompassing Caucasians, African Americans, Hispanics, Asians and American Indians. The list of peptides predicted to induce Ag-specific CD4^+^ and CD8^+^ T cell responses are as shown in Supplementary Figure 1B.

### Single peptides induced activation of CD4^+^ and CD8^+^ in HD PBMCs

**Figure 1:**
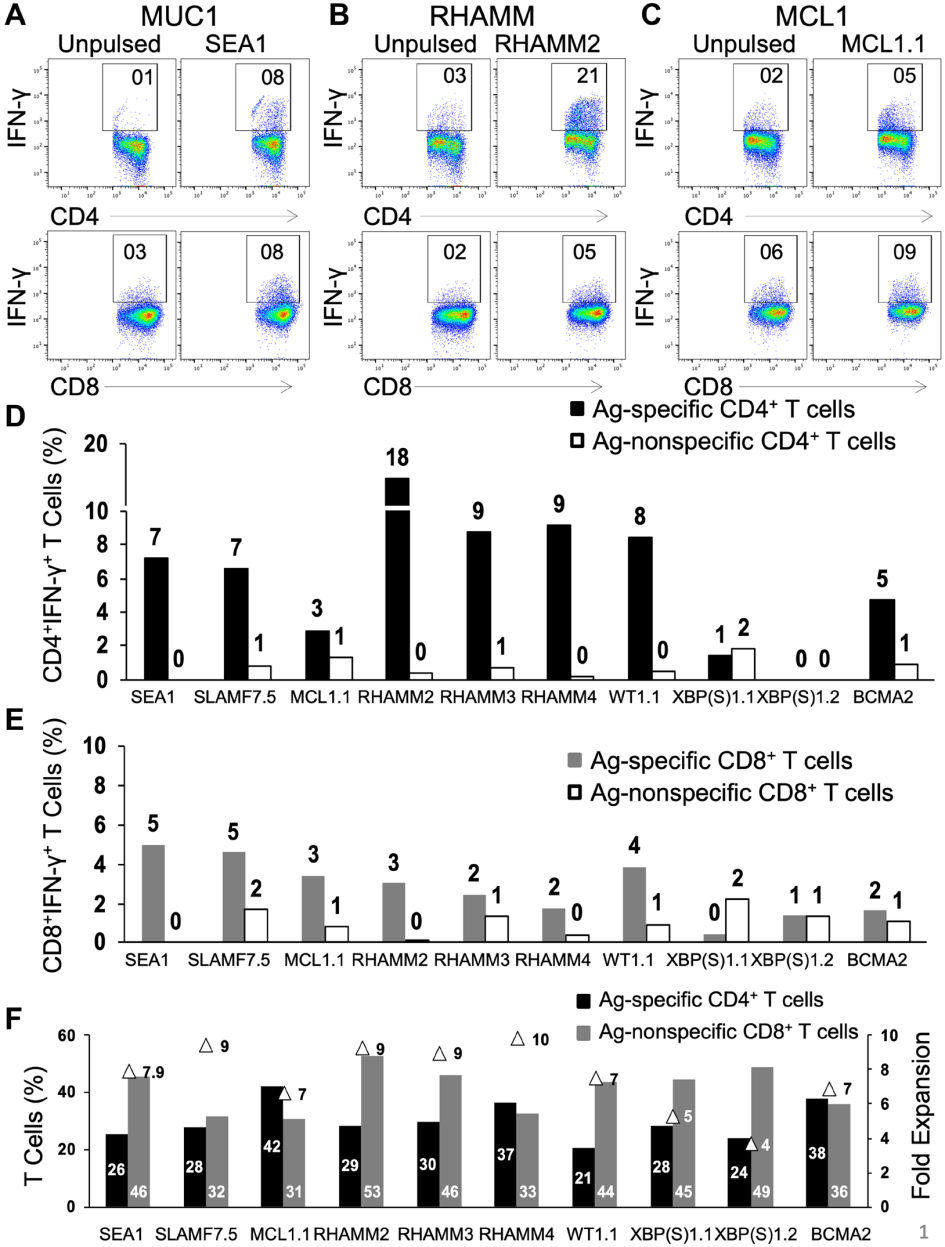
Natural CD4^+^ and CD8^+^ T cells from unfractionated healthy donor PBMCs are activated and readily propagated by peptides in an Ag-specific manner. Freshly thawed PBMCs from healthy donors were exposed to single peptides (50 μg/ml) in the presence of GM-CSF and Toll-like receptor agonists (resiquimod and LPS) followed by γ_c_ cytokine IL-7. Representative dot-plots for CD4^+^IFN-γ^+^ (top panel) and CD8^+^IFN-γ^+^ (bottom panel) following secondary stimulation of T cells generated against (**A**) SEA1 (designed from MUC1 Ag), (**B**) RHAMM2 and (**C**) MCL1.1 with either unpulsed or the specific peptide-pulsed PBMCs. Values shown for unpulsed cells are typical and have been subtracted in subsequent figures. (**D**) Bar graph depicting the percentage of Ag-specific and Ag non-specific CD4^+^IFN-γ^+^ and (**E**) CD8^+^IFN-γ^+^ T cells. (**F**) Graph depicting percentages of CD4^+^ and CD8^+^ T cells and fold expansion (triangles) observed for T cells generated following primary stimulation with SEA1, SLAMF7.5, MCL1.1, RHAMM2, RHAMM3, RHAMM4, WT1.1, XBP(S)1.1, XBP(S)1.2 or BCMA2. Data from two experiments.

We have previously reported that synthetic peptides designed from Ags were able to induce activation of CD4^+^ and CD8^+^ T cells [15]. Here the same methodology was employed to examine the ability of newly synthesized peptides to stimulate T cells *in vitro*. Briefly, unfractionated PBMCs from different HDs were stimulated with synthetic long peptides along with granulocyte-macrophage colony-stimulating factor (GM-CSF) and toll-like receptor (TLR) agonists 4 and 8 to activate innate immune cells. During the culture period that lasted for 19 days, T cell proliferation and survival were supported with interleukin-7 (IL-7), a T cell growth factor. In Figure 1, the HD PBMCs were stimulated on day 1 with the peptides from the following Ags: MUC1 (SEA1), RHAMM (RHAMM2, 3 and 4), MCL1.1, SLAMF7.5, WT1.1, XBP(S)1.1, 1.2 and BCMA2. The numbers, for e.g. RHAMM2, 3 and 4, indicate that the peptides were designed from different regions of the same Ag, here RHAMM. At the end of the culture period, T cells were harvested for secondary stimulation with PBMCs that were either unpulsed or pulsed with Ags similar or dissimilar from that used for primary stimulation to examine intracellular interferon-gamma (IFN-γ) expression in CD4^+^ and CD8^+^ T cells. Secondary stimulation of CD4^+^ T cells with unpulsed PBMCs resulted in IFN-γ expression that served as background (Figure 1A–1C, left panel). The re-stimulation of T cells with PBMCs pulsed with the specific peptide (SEA1, RHAMM2 or MCL1.1) used for primary stimulation led to robust IFN-γ expression in CD4^+^ T cells for MUC1 and RHAMM (Figure 1A–1C, top panel) and CD8^+^ T cells (Figure 1A–1C, bottom panel). In general, the response of CD4^+^ T cells was stronger than that of CD8^+^ T cells. The IFN-γ expression by CD4^+^ (Figure 1D) and CD8^+^ (Figure 1E) T cells was calculated by subtracting the background unpulsed value from that observed following restimulation with the specific peptide. The secondary exposure of T cells with an Ag different from that used for primary stimulation was considered as a negative control that is indicative of cross-reactivity. The stimulation of PBMCs with different peptides increased the total number of T cells (fold expansion) as well as enlarged both CD4^+^ and CD8^+^ T cell subsets (Figure 1F).

Amongst the Ags depicted here, MUC1, SLAMF7, RHAMM, WT1 and BCMA showed a robust Ag-specific T cell response. As against this, the peptides designed from MCL1 and XBP(S)1 gave a lower level of Ag-specific T cells. Overall, most of the novel peptides successfully induced Ag-specific CD4^+^ and CD8^+^ T cell responses. The efficacy of all peptides designed from different Ags was tested. The peptides that reproducibly activated naïve CD4^+^ and CD8^+^ T cells in an Ag-specific manner were selected to assemble peptide cocktails consisting of three or five peptides. The four peptide cocktails employed in the ensuing studies are depicted in Supplementary Figure 2. Immunization with multiple peptides can alleviate immune editing and dependency on a single Ag. Although the presence of multiple Ags in one mixture can lead to Ag competition, our studies showed that combinations can be devised that lead to strong activation of T cells to multiple peptides.

### Peptide cocktails generate Ag-specific CD4^+^ and CD8^+^ T cells from PBMCs isolated from MM patients or HDs

**Figure 2:**
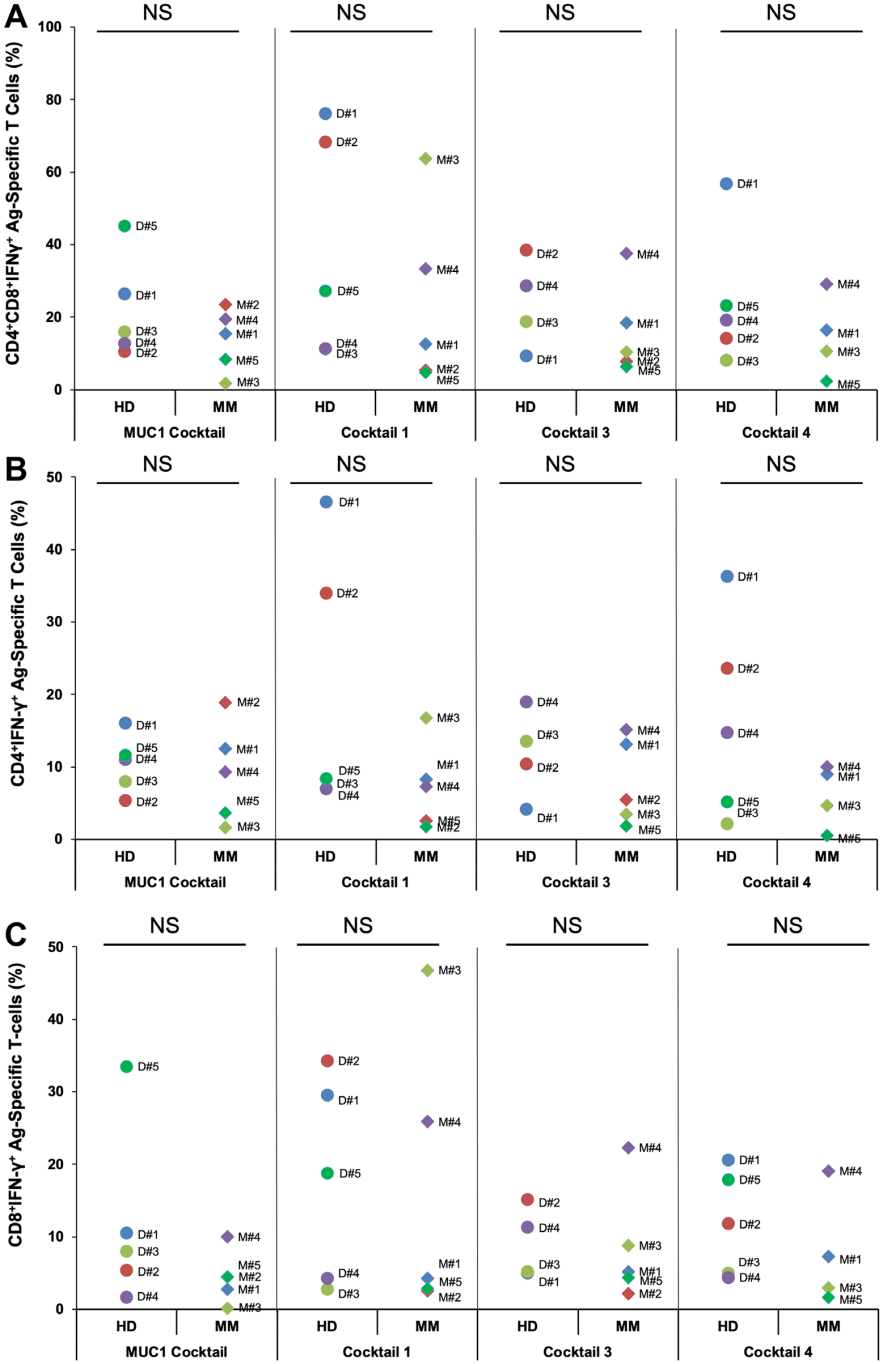
PBMCs from healthy donors or multiple myeloma patients generated Ag-specific T cells following stimulation with four different peptide cocktails designed from various antigens. PBMCs from healthy donors (HD) or multiple myeloma (MM) patients’ bloods (100 ml) were stimulated with 4 different cocktails, each consisting of either 3 or 5 peptides at 25 μg/ml for each peptide. Cells were harvested on day 19. Shown are percentages of (**A**) Ag-specific CD4^+^IFN-γ^+^ + CD8^+^IFN-γ^+^ and (**B**) CD4^+^IFN-γ^+^ and (**C**) CD8^+^IFN-γ^+^ T cells for HDs and MM patients observed following secondary stimulation with PBMCs pulsed with specific peptides present in MUC1 cocktail, cocktail 1, cocktail 3 and cocktail 4 at the end of the culture period (D19). Cocktails 3 and 4 lack MM2 and MM5 due to unavailability of cells. No statistically significant differences (NS) were observed between the different cocktails or between the HDs and MM patients in each cocktail (Student’s *t*-test *p* > 0.1 in every comparison).

To assess the ability of compiled peptide cocktails Mucin1 Cocktail (MUC1 CT), Cocktail 1 (CT1), Cocktail 3 (CT3) and Cocktail 4 (CT4) to stimulate naïve T cells, PBMCs from HDs as well as MM patients that were at different disease stages were employed in the study. PBMCs were exposed to different cocktails. The T cells harvested on day 19 were restimulated with a single peptide, which corresponded to each peptide that was present in the cocktail. Overall, as seen with HD PBMC, the peptide cocktails induced proliferation of Ag-specific CD4^+^ and CD8^+^ T cell responses following stimulation of PBMCs from MM patients, indicating the functional status of the immune system regardless of the presence of the disease (Figure 2). The differing levels of responses to various peptides may be due to different HLA types of the individuals tested. No statistically significant differences were observed between the different cocktails or between the HDs and MM patients in each cocktail (Student’s *t*-test *p* > 0.1 in every comparison).

### Subset evaluation indicated similar results for HD and MM patients

**Figure 3:**
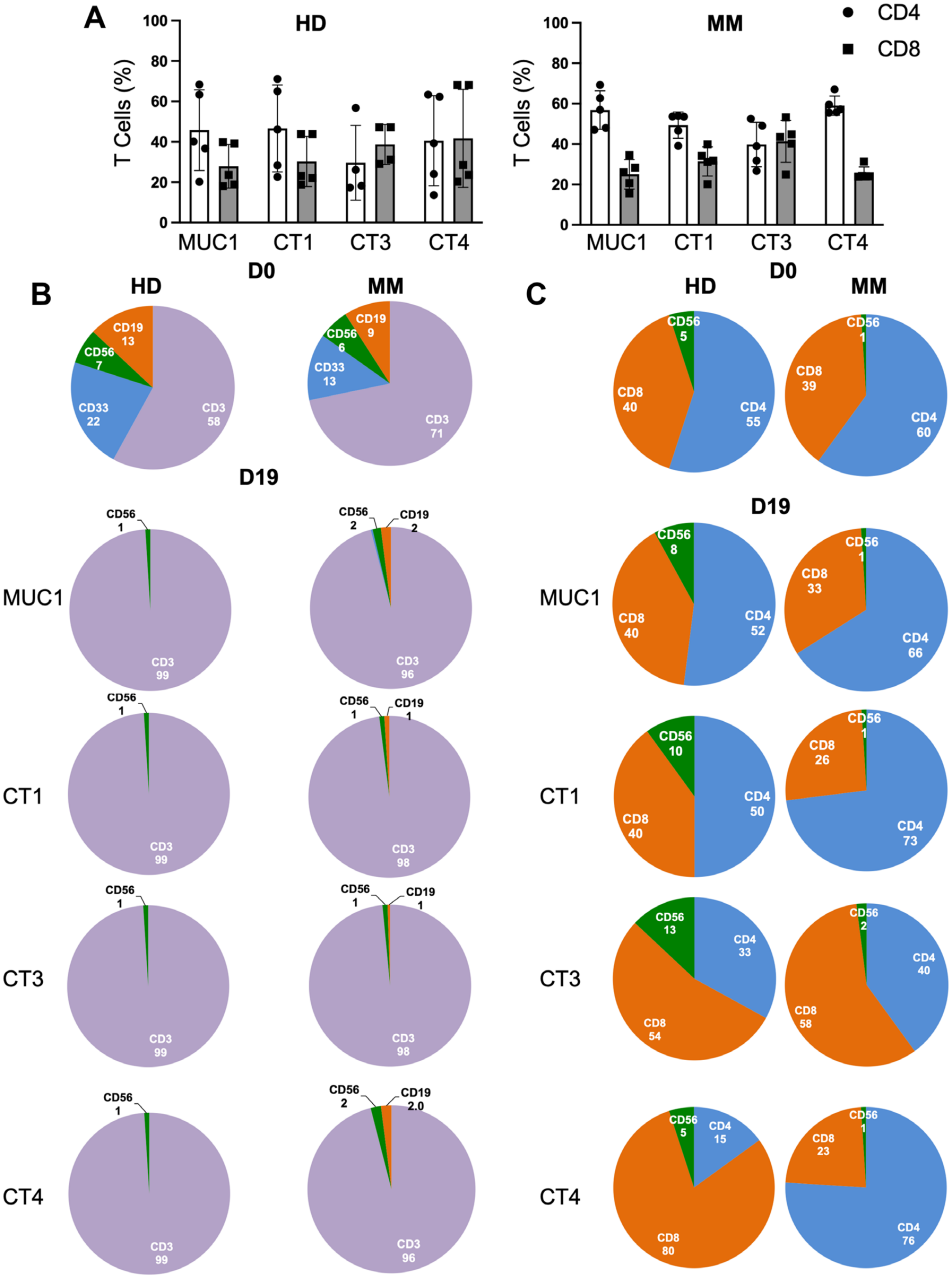
Stimulation with peptide cocktails enriches T cells equivalently regardless of the disease status. (**A**) Depiction of percentages of CD4^+^ (white) and CD8^+^ (grey) T cells at the end of culture period for 5 HDs (left panel) and 5 MM patients (right panel). (**B**) Pie charts showing percentages of immune cell subsets on D0 or D19 at end of culture period of PBMCs of HD (left panel) and MM patient (right panel) with MUC1 cocktail and cocktails 1, 3, and 4. CD19 (orange), CD56 (green), CD33 (blue) and CD3 (purple) are shown. CD3+ population on D19 was always greater than 85% positive. (**C**) CD3^+^ T cells were further analyzed for CD4^+^ (blue), CD8^+^(orange) and CD56^+^ (green) for HD (left panel) and MM patient (right panel). Percents of CD4^+^ and CD8^+^ T cells depended upon the particular cocktail used for primary stimulation and the HLA genotype of the individual. Data were similar for all ten samples. No statistically significant differences were observed (Student’s *t*-test). Representative data are shown.

The stimulation of PBMCs with different peptides increased the total number of T cells (fold expansion) as well as enlarged both CD4^+^ and CD8^+^ T cell subsets (Figure 3A). The day 0 PBMCs and cells harvested on day 19 were examined for the levels of different cell populations, such as CD3 (T cells), CD33 (myeloid cells), CD56 (Natural Killer Cells, NK cells) and CD19 (B cells). The 19 day culture resulted in a large expansion of CD3^+^ T cells, from about 50% at day 0 to greater than 90% on day 19 in all of the cocktails, whereas the CD33, CD56 and CD19 cell percentages decreased greatly (Figure 3B). Analysis of the CD3^+^ T cells showed the majority were either CD4^+^ or CD8^+^, with the actual percentages varying depending upon the cocktail used for stimulation (Figure 3C). There was a smaller percentage of CD3^+^CD56^+^ NKT cells, usually 10% or less. There were no statistically significant differences between the percentages of the different cell populations of MM patients and HDs. The data shown are representative of the samples studied.

### Generation of CD4^+^ and CD8^+^ effector (T_EM_) and memory (T_CM_) from MM patients and HDs at the end of the culture period

**Figure 4:**
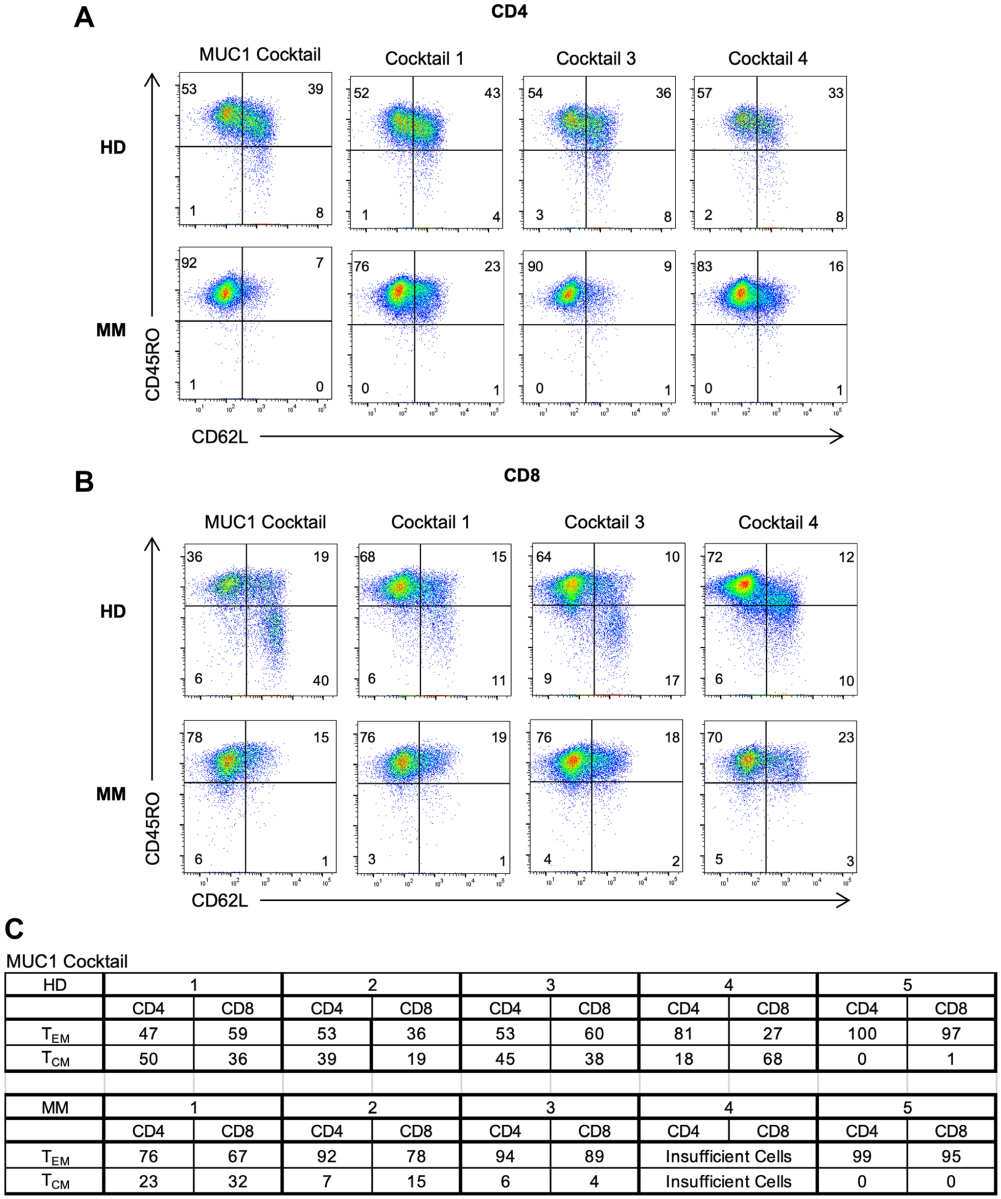
Generation of both effector and memory T cells in MM patients and HDs following peptide activation. Flow cytometry dot plots depicting (**A**) CD4^+^ and (**B**) CD8^+^ T_EM_ (CD45RO^+^CD62L^-^) and T_CM_ (CD45RO^+^CD62L^+^) for HD and MM on D19 following stimulation of multipeptide cocktails. Representative data shown for 2 individuals. (**C**) Chart showing composite results of T_EM_ and T_CM_ for MUC1-activated HD (top) and MM patients (bottom). Statistical analysis indicated no significant differences (Student’s *t*-test).

The T cells harvested at the end of the culture period were stained with CD62L and CD45RO for phenotypic classification [30]. All four peptide cocktails generated CD4^+^ and CD8^+^ T_EM_ (CD45RO^+^CD62L^-^) and T_CM_ (CD45RO^+^CD62L^+^) from PBMCs from HDs as well as MM patients (Figure 4A, 4B). Overall, it seems that MUC1-activated PBMCs from HDs generated CD4^+^ T_CM_ (3/5) to a greater extent than T_EM_ (2/5) whereas PBMCs from MM patients induced CD4^+^ T_EM_ to a higher level than T_CM_. However, in the case of CD8^+^ T cells, the propensity to develop T_EM_ was greater than T_CM_ in both HDs as well as the MM patients (Figure 4C). T_CM_ cells are more likely to survive and establish immunologic memory. Statistical analysis indicated no significant differences (Student’s *t*-test). The data for one HD and one MM patient are shown (Figure 4A, 4B). The Supplementary Figure 4 depicts the T_EM_ and T_CM_ observed in five different HDs and MM patients for CT1 (Supplementary Figure 3A), CT3 (Supplementary Figure 3B) and CT4 (Supplementary Figure 3C).

We further examined the expression of CD69 and CD103, the receptors used to delineate tissue resident memory cells (T_RM_) [31, 32]. These cells are thought to develop from killer cell lectin-like receptor G1 negative (KLRG1^-^) memory precursor cells and are crucial for peripheral immunity against re-infection and cancer [33, 34]. CD8^+^ T cells expanded from either HD or MM PBMCs showed expression of CD69 and CD103 (Supplementary Figure 4A, 4B). CD122, the shared IL-2 and IL-15 receptor-β chain, is essential for the maintenance of T_EM_ and T_CM_ and for the development of T_RM_ cells [35]. Its expression varied and was dependent on the peptide cocktail. Furthermore, both CD4^+^ and CD8^+^ T cells expressed CD122 (Supplementary Figure 4C, 4D). However, CD122 was augmented to a greater level on CD8^+^ T cells from HD or MM patients in response to different peptide cocktails. The cells also showed an increase in the accumulation of neutral lipids, chemokine receptors (CD49a, CXCR6, CD101 and CXCR3) and transcription factors (Notch1) (data not shown).

### Multiclonal expansion of Ag-specific CD4^+^ and CD8^+^ T cells with cytolytic abilities

**Figure 5:**
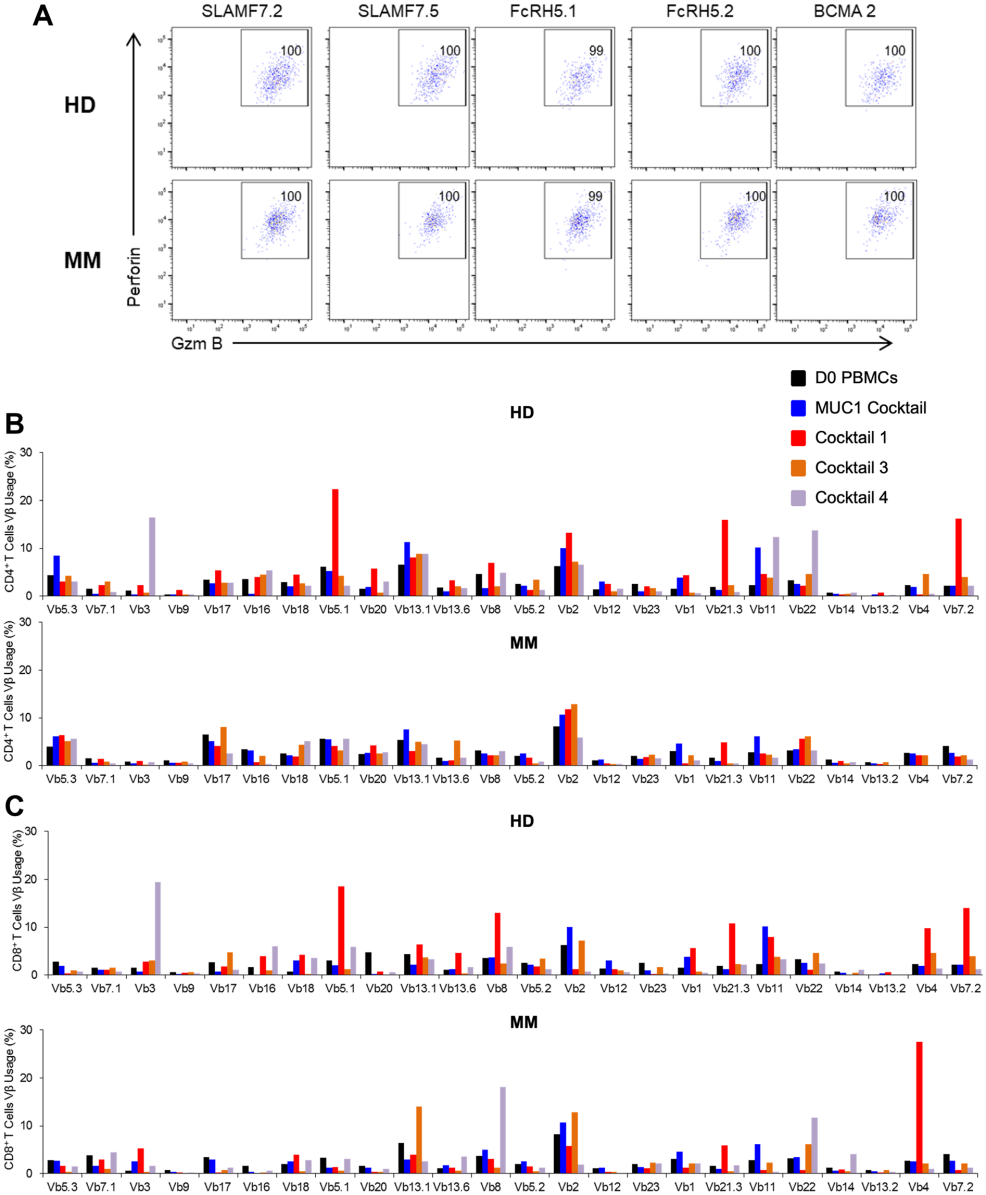
Functional characterization following stimulation to peptide cocktails leads to multiclonal expansion of Ag-specific CD4^+^ and CD8^+^ T cells possessing cytolytic capabilities at the end of the culture period (D19). Dot-plot showing expression of perforin and granzyme B on (**A**) CD8^+^ T cells in HD1 (top panel) and MM1 (bottom panel) following restimulation with each peptide from CT3. T cell receptor (TCR) Vβ repertoire based on flow analysis of (**B**) CD3^+^CD4^+^ and (**C**) CD3^+^CD8^+^ of HD1 or MM1 harvested on day 19. Representative data are shown and statistical analysis indicated no significant differences among the ten samples analyzed (Student’s *t*-test).

The cytolytic ability of CD4^+^ and CD8^+^ T cells was determined by examining the expression of perforin and granzyme B. The gating strategy employed is portrayed in Supplementary Figure 5. Briefly, the CD3^+^CD4^+^ and CD3^+^CD8^+^ T cells were gated on cells expressing IFN-γ, which were further analyzed for perforin and granzyme B positivity. More than 90% of the IFN-γ^+^ cells stimulated by all of the cocktails were positive both for perforin and granzyme B, proteins that are surrogates of lytic activity (Figure 5A and 5B and data not shown).

Next we assessed the TCR diversity exhibited by the naïve and activated CD4^+^ and CD8^+^ T cell populations at the initiation and commencement of the culture period. All five HDs and MM patients exhibited an increase in the number of both T cell types. Both CD4^+^ and CD8^+^ T cells exhibited multiclonal expansion regardless of the PBMC source. Some clones showed more extensive proliferation than others. A representative example, (Figure 5B and 5C), suggests that the peptide cocktails successfully induced a polyclonal response with a few dominating clones in both CD4^+^ and CD8^+^ T cell compartments, regardless of the source of the PBMCs. In some cases, a monoclonal response has been observed (data not shown).

### Comparative metabolic profiles were observed in MM patients and HDs

**Figure 6:**
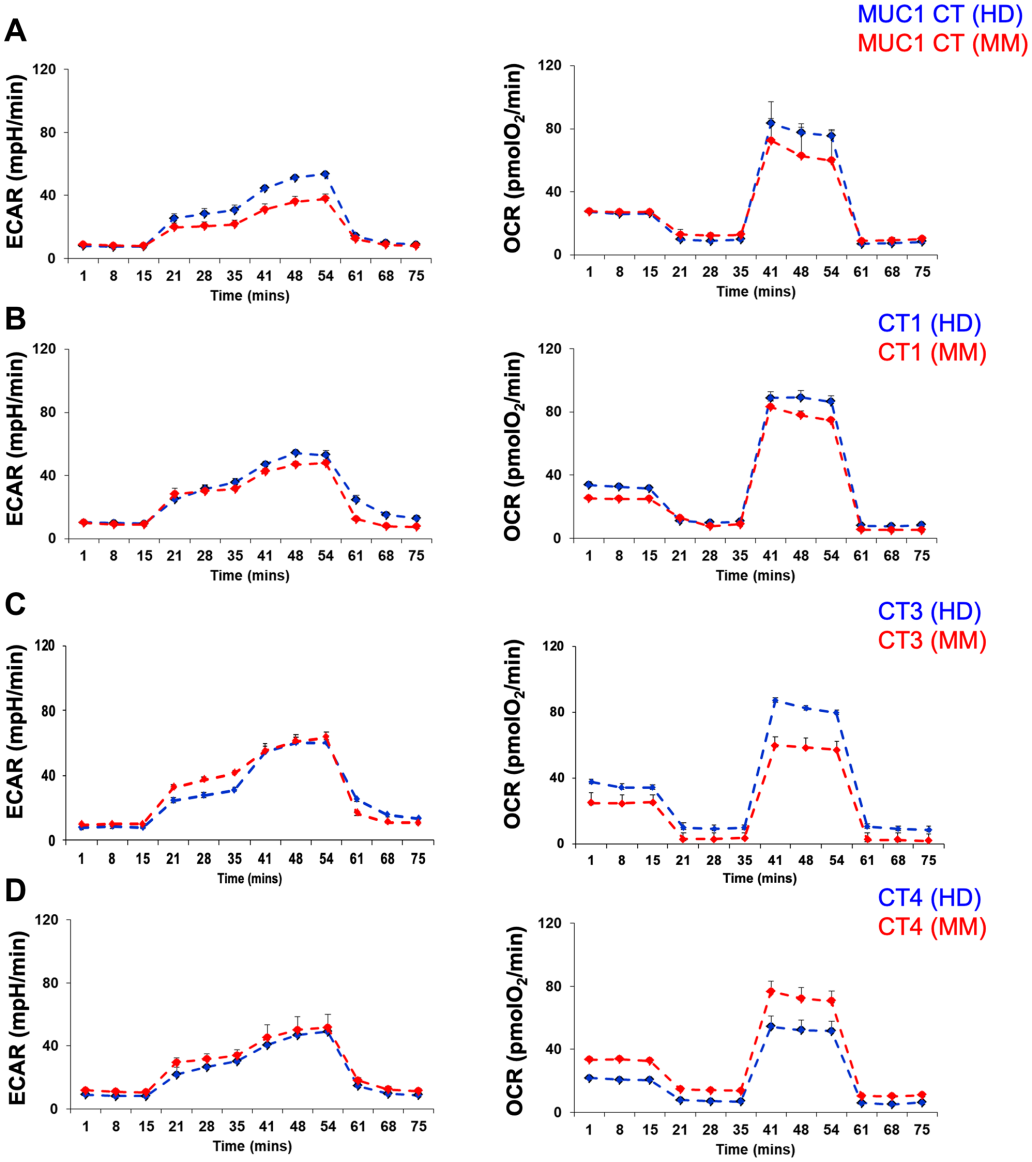
Metabolic profile of healthy donor or multiple myeloma patient’s memory T cell population varies depending upon the peptide cocktail used for stimulation. Glycolysis stress test was conducted to examine the extracellular acidification rate (ECAR) in response to glucose, oligomycin, and 2-deoxy-D-glucose (2DG). The Mito-cell stress test assessed the oxygen consumption rate (OCR) following treatment with oligomycin, carbonyl cyanide-p-trifluoromethoxyphenylhydrazone (FCCP) and rotenone/antimycin. The histograms for ECAR (left panel) and OCR (right panel) are depicted for T cells generated following exposure of PBMCs from healthy donor (blue histogram) or multiple myeloma patient (red histogram) to MUC1 cocktail (**A**), cocktail 1 (**B**), cocktail 3 (**C**) and cocktail 4 (**D**). Representative data are shown and no statistically significant differences were observed among the six samples analyzed (Student’s *t*-test).

T cells at the tumor site in MM patients have been shown to be exhausted [36]. Increasing evidence suggests altered cellular metabolism to be one of the hallmarks of tumor cells. Furthermore, studies in the past have shown a positive association between metabolic disorder and MM incidence. Therefore, to understand the metabolic profile of the culture generated T cells, we first assessed the ability of PBMCs from HDs or MM patients to induce glycolysis (ECAR, extracellular acidification rate) or oxidative phosphorylation (OXPHOS, oxidative phosphorylation). At the end of the culture period (day 19), the expanded cells following exposure to different peptide cocktails (MUC1 Cocktail or Cocktails 1, 3 or 4) were harvested. The metabolic profile for a representative HD and MM patient is depicted in Figure 6. The rate of glycolysis was similar in cells expanded from HDs or from MM patients following stimulation with either MUC1 Cocktail (Figure 6A, left panel), Cocktail 1 (Figure 6B, left panel), Cocktail 3 (Figure 6C, left panel) or Cocktail 4 (Figure 6D, left panel). The rate of OXPHOS was also equivalent in T cells derived from PBMCs of HD compared to that from MM patients regardless of the peptide cocktail (Figure 6A–6D, right panel). Overall, the extent to which glycolysis and OXPHOS were activated in different MM PBMCs was comparable to that of HDs with no statistical differences noted (Student’s *t*-test, *p* > 0.1 in all comparisons). Further calculation revealed that basal respiration, ATP production, maximal respiration, spare respiratory capacity (SRC) and non-mitochondrial-derived OCR were observed to be similar in the MM-derived T cells to those from HDs (data not shown), suggesting that the metabolic profile of MM-derived day 19 cells is similar to those of HD cells following peptide-driven stimulation in the *ex vivo* culture. No statistical differences were observed.

## DISCUSSION

Despite the development of novel immunotherapeutic agents, MM remains largely an incurable disease. Based on unprecedented response rates, anti-BCMA CAR-T cells have been recently approved for the treatment of MM, although the observed median progression free survival was 8.8 months and patients eventually relapsed [37, 38]. The loss of target Ag has been observed in few cases, and we postulate that targeting multiple Ags would prevent tumor escape. Peptide cocktails designed from XBP(S)1, CD138 and CS1 have been shown to generate effector memory responses aginst HLA-A2^+^ MM cells [8]. Lulla et al. have developed T cells by stimulating patient PBMCs with autologous dendritic cells (DCs) loaded with 15 mer overlapping peptides designed from PRAME, SSX2, MAGEA4, NY-ESO-1 and Survivin [39]. These T cells were safely administered to MM patients and exhibited persistence. Our protocol to generate CD4^+^ and CD8^+^ T_EM_ and T_CM_ responses is HLA-independent which would be an advantage over existing methods [15].

It has proved daunting to generate therapeutically active tumor infiltrating lymphocytes (TILs) from tumor types other than melanoma. Furthermore, it has not been possible before now to expand equivalently performing T cells from blood draws [40]. In marked contrast to other described methods, our patented culture involves minimal cell manipulation, is accomplished in 19 days, generates therapeutic quantities of activated effector and central memory T cells, and uses unfractionated PBMCs that are readily available from patients. Two days of exposure of PBMCs to GM-CSF plus paired Toll-like receptor agonists (resiquimod and lipopolysaccharide – LPS) to simulate a life-threatening infection, results in upregulated co-stimulatory molecules and CD11c expression on the CD33^+^ myeloid subpopulation, which is capable of processing synthetic long peptides from tumor-associated Ags. Seventeen additional days of exposure to exogenous IL-7 produces sustained expansion of interferon-γ-producing, Ag-specific CD4^+^ and CD8^+^ T cells with minimal production of regulatory T cells (T_reg_ cells) [15]. Our culture successfully drives large numbers of effector and central memory CD4^+^ and CD8^+^ T cells from healthy donors and cancer patients, including MM, breast and pancreas patients, in contrast to CAR-T cells that are comprised of effector rather than memory cells. CAR-T cells are limited by the requirement of a cell surface antigen, whereas our natural T cells make use of natural major histocompatibility complex restrictions to discern what is going on in cancer cell targets. Our natural, non-genetically modified, T cells retain their physiological signaling mechanisms, unlike genetically modified CAR-T cells that exhibit excessive, abnormal signaling of the T cell caused by the chimeric receptors’ tonic co-stimulation, which routinely results in cytokine release syndrome (CRS) in patients. This has not been reported in ACT.

In this study, we employed artificial neural algorithms to design peptides from prevalently expressed Ag in MM. The HD PBMCs, when stimulated with single peptides from MUC1, MAGEA6, CT45, SEPT9, SLAMF7 (CS1), FcRH5, BCMA, RHAMM and NY-ESO-1 reproducibly generated Ag-specific CD4^+^ and CD8^+^ effector and memory T cells. These peptides, when combined in a cocktail, successfully activated naïve T cells from PBMCs from MM or HDs and differentiated CD4^+^ and CD8^+^ T cells into multiclonal T_EM_ and T_CM_. Generation of memory T cells is vital for efficient tumor control, as more-differentiated effector T cells may downregulate lymphoid homing molecules, receptors to access homeostatic cytokines, and may enter proapoptotic and senescent states [41]. It was generally observed that CD4^+^ T cells were in greater number compared to CD8^+^ T cells. However, Cocktail 3 generated a higher proportion of CD8^+^ T cells compared to CD4^+^ T cells regardless of the PBMC source. On secondary stimulation with specific peptides, T cells expressed IFN-γ, granzyme B and perforin, suggesting lytic capabilities. The CD4^+^ and CD8^+^ memory T cells expressed different chemokine receptors, thereby opening up the possibility of these cells trafficking to the solid tumor microenvironment [42]. CD8^+^ T cells expressing CD69 and/or CD103, indicative ot T_RM_, were observed. These cells expressed different key indicators of T_RM_: chemokine receptors CCR6 and CXCR3 and transcription factors such as Notch1 [43, 44].

Recent advances in metabolic profiling suggest that metabolic reprogramming is absolutely essential for proper functioning of immune cells [45]. The switch from OXPHOS to aerobic glycolysis is critical for cell proliferation and synthesis of various cell subunits. Furthermore, the metabolic shift to OXPHOS and fatty acid oxidation supports cell survival for memory T cells [46]. It was observed that the *in vitro*-generated T cells from either MM patients or HDs were capable of inducing glycolysis and OXPHOS. The extent to which these pathways were stimulated varied amongst different cocktails, suggesting that these cells are metabolically sufficient to undertake different responsibilities, given the strong activation evoked by the *ex vivo* culture. It would be important to understand whether peptide affinity and avidity or disease state regulated the stimulation of metabolic pathways.

Several ongoing clinical trials are assessing the efficacy of CAR-T cells expressing SLAMF7 (CS1) and BCMA [47]. A phase I study employing DFRF4539A, an anti-FcRH5 antibody-drug conjugated to monomethyl auristatin E, was ineffective in the treatment of patients with relapsed or refractory MM [48]. However, clinical trials examining the efficacy of bispecific antibodies, GO39775 targeting FcRH5/CD3, are ongoing [49]. Peptide vaccination with RHAMM-R3 peptide induced an immunological and clinical response in MM patients [50]. Based on the data presented here, it would be worthwhile to consider the novel peptides designed here for Ags overexpressed in MM (MUC1, SLAMF7, BCMA, FcRH5, RHAMM) or cancer testis Ags (CT45, NY-ESO-1) and SEPTIN9 as potential targets for MM immunotherapy.

This *ex vivo* culture system reproducibly gives rise to high frequencies of Ag-specific, T1-type T cells, both CD4^+^ and CD8^+^, generated from PBMCs from MM patients as well as from unvaccinated donors. Overall, the different cellular phenotypic, functional, and metabolic parameters were found to be similar between the MM patient and HD PBMC-derived T cells following peptide-driven culture stimulation. The long peptides used for stimulation were processed and presented by antigen presenting cells derived from the PBMCs. The identification of multiple Ags, present on MMs as well as most solid cancers, provides evidence that this technology can be expanded to most cancer patients. We have already shown that this culture method can be scaled up to generate therapeutic doses of activated T cells for ACT. We have also confirmed that similarly activated T cells can be elicited from PBMCs from breast and pancreas cancer patients. Given the likely immune suppressive environment of tumors, co-treatment with checkpoint inhibitors may substantially potentiate T cell survival and expansion post-infusion.

These results provide the basis for an additional novel immunotherapy, ACT, for MM patients, all of whom relapse from available therapies. In our study, the successfully activated CD4^+^ and CD8^+^ T cells from PBMCs comprised both effector (T_EM_) and central memory (T_CM_) cells from HDs and MM patients. Generation of memory cells adds an important dimension to the effector cells generated by CAR-T cell therapy. Future studies will determine if these cells engraft in their hosts. Expression of granzyme B and perforin in > 90% of the IFN-γ^+^ T cells suggests cytolytic activity. This study provides evidence that the novel peptides can be used for a Phase I clinical trial in MM that will assess the toxicity, engraftment, persistence, and expansion of adoptively transferred T cells. A First-in-Human study is in late planning stages for relapsed/refractory MM, a tumor for which cellular therapy has been initially effective.

## MATERIALS AND METHODS

Isolation and preservation of PBMCs: The collection and preservation of HD and MM patient PBMCs were performed as described [15]. Briefly, leukapheresis was performed as per the guidelines compiled by the American Association of Blood Banks on 5 healthy volunteers with their consent. Samples were subjected to Ficoll-Hypaque density separation (Ficoll-Paque Plus, Thermo-Fisher #17-1440-02). For cancer patients, 100 ml of whole blood was collected by peripheral venipuncture which was then purified for PBMCs by the Ficoll-Hypaque density gradient centrifugation (2000 rpm for 20 minutes). For this study, PBMCs were collected from 5 MM patients at different stages of cancer. The cells were cryopreserved in liquid nitrogen using either 10% dimethyl sulfoxide (DMSO; Sigma #02650) or Cryostar CS10 (BioLife Solutions #210374).

Patient Characteristics are as follows: MM1 has smoldering MM, with M spike increasing rapidly. No prior treatment. MM2 has amyloidosis and smoldering MM, off therapy for 6 years. Patient had received an autologous bone marrow transplantation 6 years prior. MM3 has MGUS, with type 2 diabetes. MM4 is a 70 year old male with MM International Staging System (ISS) 2 for one year prior to blood collection. He received lenalidomide 3 weeks prior and bortezomib and dexamethasone one week prior. MM5 has untreated smoldering MM.

### Peptide design and synthesis

The peptides were mapped using open access discovery software, the NetMHCpan servers 3 and 3.2 that predicts MHC I (9 mer) & II (15 mer) binding hotspots, respectively, based on artificial neural networks. The method of designing peptides is discussed in reference 15. Briefly, the Fasta sequence of the protein was submitted to NetMHCpan server 3 for determining MHC I hotspots. The alleles that were employed to detect the hotspots are as follows:

HLA-A01:01,HLA-A02:01,HLA-A03:01,HLA-A11:01,HLA-A24:02,HLA-B07:02,HLA-B08:01,HLA-B15:01,HLA-B40:01,HLA-B44:02,HLA,B51:01,HLA-C03:03,HLA-C03:04,HLA-C04:01,HLA-C05:01,HLA-C06:02,HLA-C07:01,HLA-C07:02,HLA-A23:01,HLA-A25:01,HLA-A26:01,HLA-A29:02,HLA-A32:01,HLA-B14:02,HLA-B18:01,HLA-B44:03,HLA-B53:01,HLA-B57:01,HLA-C02:02,HLA-C08:02,HLA-A30:01,HLA-A30:02,HLA-A33:03,HLA-A34:02,HLA-A68:02,HLA-A74:01,HLA-B15:03,HLA-B58:01,HLA-C16:01,HLA-B35:01,HLA-B42:01,HLA-B45:01,HLA-B49:01,HLA-C17:01,HLA-C18:01,HLA-C01:02,HLA-A31:01,HLA-A68:01,HLA-B52:01,HLA-C08:01,HLA-C12:03,HLA-A02:03,HLA-A02:06,HLA-A02:07,HLA-B13:01,HLA-B15:02,HLA-B35:01,HLA-B38:02,HLA-B40:02,HLA-B46:01,HLA-B55:02,HLA-B54:01,HLA-C03:02,HLA-C14:02

The peptide length was adjusted to 9 and the threshold for strong and weak binders was adjusted to 0.5 and 2, respectively. In the output data, the last column denoted the hotspots for MHC I binding.

To delineate the MHC II hotspots, we NetMHCpan 3.2 was utilized. After inserting the Fasta sequence, the allele information was added, which were: DRB1_0101,DRB1_0102,DRB1_0301,DRB1_0302,DRB1_0401,DRB1_0403,DRB1_0404,DRB1_0405,DRB1_0407,DRB1_0701,DRB1_0802,DRB1_0803,DRB1_0804,DRB1_0901,DRB1_1001,DRB1_1101,DRB1_1104,DRB1_1201,DRB1_1201,DRB1_1301,DRB1_1302,DRB1_1303,DRB1_1401,DRB1_1404,DRB1_1406,DRB1_1501,DRB1_1502,DRB1_1503,DRB1_1602. In this case, the peptide length was restricted to 15 and the threshold for strong and weak binders was adjusted to 2% and 10%, respectively. The last column of the output file indicated the MHC II hotspots. The peptides were designed where both MHC I and II hotspots overlapped.

The designed peptides have high affinity for multiple class I & II haplotypes expressed in individuals across different races and ethnicities. Processing of long peptides (17 to 41 amino acids) was essential for activation of naive T cells [15]. The peptides were manufactured by Mayo Peptide Synthesis Laboratory or GeneMed (MUC1 peptides).

### Generation of T cells and restimulation for functional analysis

PBMCs were cultured and restimulated as described [15]. Briefly, PBMCs were thawed on day 0 (D0). After washing the cells, a density of 6 × 10^6^ cells/ml was resuspended in AIM-V media (Gibco #0870112-DK) with 0.5% human AB serum (HuAB, Gemini Bioproducts #100-512) and 80 ng/ml GM-CSF (R & D #215-GMP-010). 0.5 ml of cell suspension was added per well in a 48-well cluster plate. On D1, the cells were stimulated (0 h) with a single peptide (50 μg/ml) or a cocktail of peptides (25 μg/ml for each peptide). Resiquimod (R848, 6 μg/ml-Invivogen #vac-r848) and LPS (1 ng/ml-Invivogen #vac-3pelps) were added after 4 h and 4.5 h, respectively, after Ag pulsing. On D2 of culturing, the cells were detached by washing with Ca^+2^/Mg^+2^-free PBS (Gibco #10010-23) and harvested. These cells were resuspended in 6 ml of AIM-V media containing 2% of HuAB serum and 50 ng/ml of IL-7 (Miltenyl #130-095-364). 2 ml of cell suspension were added to each well of fresh 24-well cluster plates. The cells were harvested on D19 with intermittent splits on D8, D12 and D15.

A secondary stimulation was performed with the harvested T cells to analyze phenotypic and functional characteristics. For this, another PBMC vial was thawed (D17) and stimulated (D18) with each cocktail peptide (50 μg/ml) singly in the presence of Amphotericin B (125 ng/ml) (Lonza #17-836E). On D19, the harvested T cells and aforesaid Ag-pulsed PBMCs were co-cultured overnight at a density of 2:1, which were then used for analysis. For assessing intracellular IFN-γ, monensin (GolgiStop, BD Biosciences, San Diego CA, USA, #554724) was added after 4–6 h to block the export of endogenously produced cytokines. The cells were then surface stained for CD4 and CD8 followed with intracellular staining for IFN-γ.

### Metabolic assay

Seahorse XFe bioanalyser was used to measure the Extracellular Acidification Rate (ECAR) and Oxygen Consumption Rate (OCR). The assay was performed as follows: Seahorse 96 well plates were first coated with Cell-Tak (Corning #354240) for 20 minutes. In the meantime, the cells were resuspended in Seahorse XF base DMEM media with (Agilent Technologies #103334-100) and without phenol red (Agilent Technologies #103335-100). For OCR analysis, the media contained glucose (10 mM; Sigma #G5146), sodium pyruvate (1 mM) and glutamine (2 mM) whereas for ECAR the media had only glutamine (2 mM). Day 19 cells (1.2 × 10^5^) were added to each well (3 wells per sample). The cells were spun down and then placed in a non-CO_2_ incubator at 37°C for 1 h. ECAR and OCR analyses were conducted under basal conditions and after adding the following reagents: ECAR assessment-glucose (10 mM), oligomycin (1 μM; Sigma #O4867-5 mg), 2-deoxy-D-glucose 2-DG (5 mM) and for OCR-oligomycin (1 μM), carbonyl cyanide-p-trifluoromethoxyphenylhydrazone (FCCP) (1 μM; Sigma #C2920-10 mg), rotenone (0.5 μM; Sigma #R8875) and antimycin (5 μM; Sigma #A8674-25 mg).

### Antibodies

The following fluorochrome conjugated anti-human antibodies were used: CD3 APC efluor 780 (eBioscience #47-0036-42) or BV650 (Biolegend #317324), CD4 BV 510 (BD Horizon/BD Biosciences #562970), CD8 evolve 655 (eBioscience #86-0088-42), CD33 APC (eBioscience#17-0338-42), CD56 efluor 710 (eBioscience #46-056-42) or FITC (Biolegend #304604), PD-1 BV 785 (BioLegend #329930), CCR7 BV785 (Biolegend #353230), CD19 BV785 (Biolegend #302240), CD62L BV785 (Biolegend #304830), IFN-γ efluor 450 (eBioscience #48-7319-42), Perforin Alexa Fluor 647 (Biolegend #353322) or PE (Biolegend #353304), granzyme FITC (Biolegend #515403). Appropriate isotype control antibodies were employed to determine the specificity of test antibodies.

### Multiparameter flow cytometry

Cells were centrifuged and stained for 30 mins at RT with Live/Dead UV Blue stain (Life Technologies, # L23105; diluted 1:1000 in PBS) for assessing viability. After washing, cells were exposed to Fc receptor block (50 μg of unconjugated human IgG; Sigma Aldrich #S-8032) and stained for surface proteins (30 mins at 4°C) by adding respective antibodies in FACs buffer. The FACS buffer is Ca^+2^/Mg^+2^-free PBS with 1% heat-inactivated fetal bovine serum (Sigma Aldrich #F2442) and 0.02% sodium azide (Sigma Aldrich #S-8032). For analyzing intracellular proteins, the cells were then subjected to fixation and permeabilization according to the manufacturer’s guidelines (eBioscience, San Diego CA, USA, #00-5123-43, #00-5223-56 and #00-8333-56; BD Biosciences #51-2090KZ, #51-2091KZ) followed by staining with appropriate antibodies. Flow cytometry data was acquired on Fortessa (BD Bioscience) and analyzed with FACSDiva software (BD Biosciences) or Flowjo.

### Vβ frequency analysis

The Vβ repertoire of CD3^+^CD4^+^ and CD3^+^CD8^+^ T cells was assessed by manufacturer’s protocol with the kit – IOTest Beta Mark (Beckman Coulter). Antibodies detect only about 70% of the T cell receptor (TCR) Vβ repertoire.

### Statistical analysis

To compare the means of each of the groups or between HDs and MM patients, a two-sided Student’s *t*-test was used. *p* ≤ 0.05 was considered statistically significant.

## SUPPLEMENTARY MATERIALS



## Abbreviations

ACT: Adoptive Cell Transfer
Ag: Antigen
BCMA: B Cell maturation Antigen
CAR-T: Chimeric Antigen Receptor T Cells
CD3/4/8/33/56/19: Cluster of Differentiation 3/4/8/33/56/19
CRS: Cytokine Release Syndrome
CTAs: Cancer Testis Antigens
CT1: Cocktail 1
CT3: Cocktail 3
CT4: Cocktail 4
CT45: Cancer Testis Antigen Family 45
2-DG: 2-Deoxy-D-Glucose
D0/1/2/17/19: Day 0/1/2/17/19
ECAR: Extracellular Acidification Rate
FcRH5: Fc Receptor Like 5
FCCP: Carbonyl Cyanide-p-Trifluoromethoxyphenylhydrazone
GM-CSF: Granulocyte-Macrophage Colony-Stimulating Factor
HD: Healthy Donor
H: Hour
IFN-γ: Interferon-Gamma
IL-7: Interleukin-7
KLRG1: Killer Cell Lectin-Like Receptor G1
LPS: Lipopolysaccharide
MAGEA3/6: Melanoma Antigen Family A3/6
MCL1: Myeloid Cell Leukemia 1
MHC I: Major Histocompatibility Complex I
MHC II: Major Histocomptability Complex II
MM: Multiple Myeloma
MUC1: Mucin 1
MUC1 CT: Mucin 1 Cocktail
NK cells: Natural Killer Cells
NY-ESO-1: New York Esophageal Squamous Cell Carcinoma 1
OCR: Oxygen Consumption Rate
OXPHOS: Oxidative Phosphorylation
PBMC: Peripheral Blood Mononuclear Cells
RHAMM: Receptor for Hyaluronan-Mediated Motility
SEA Peptides: derived from Sperm Protein, Enterokinase and Agrin domain in MUC1
SEPT9: SEPTIN9
SLAMF7: Self-Ligand Receptor of the Signaling Lymphocytic Activation Molecule Family 7
SRC: Spare Respiratory Capacity
TCR: T Cell Receptor
TLR: Toll-Like Receptor
T_EM_: T Effector Memory Cells
T_CM:_ T Central Memory Cells; T_RM_: T Resident Memory Cells
T_reg_: Regulatory T cells
Vβ: Variable Beta
WT1: Wilms Tumor 1
XBP(S)1: Spliced isoform of X-Box Binding Protein 1
