# Supplementary material for: Multipeptide stimulated PBMCs generate TEM/TCM for adoptive cell therapy in multiple myeloma
Source: Oncotarget. 2021 Sep 28;12(20):2051–67. doi: 10.18632/oncotarget.28067 (PMC8487724; doi:10.18632/oncotarget.28067)
Supplement: Supplementary file 1 [file oncotarget-12-2051-s001.pdf]

Multipeptide stimulated PBMCs generate T_{EM}/T_{CM} for adoptive cell therapy in multiple myeloma

SUPPLEMENTARY MATERIALS

A

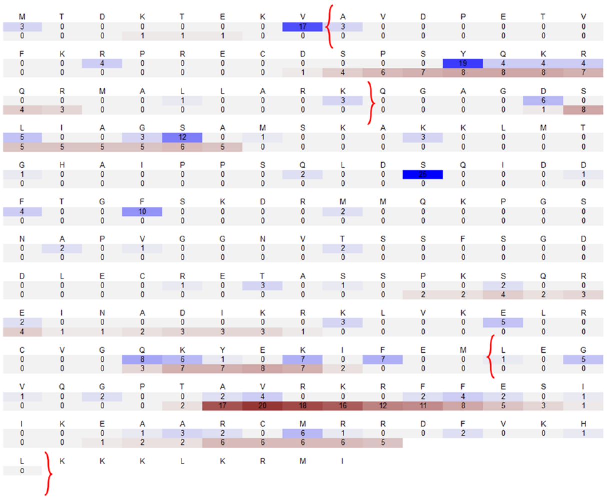

B

1. SEA1 (MUC1): SPQLSTGVSFFFLSFHISNLQFNSSLEDPSTD (32 MER)
2. SEA2 (MUC1): STDYYQELQRDISEMFLQIYKQGGFLGLSNIKFRPGSVV (39 MER)
3. SEA3 (MUC1): DVETQFNQYKTEAASRYNLTISDVSVSDVPFP (32 MER)
4. BCMA 2: FPLPAMEEGATILVTTKTNDYCKSLPAALSATEIEKS (37 MER)
5. CD38.1: YQSCPDWRKDCSNNPVSVFWKTVSRR (26 MER)
6. CD38.2: ACDVVHVMLNGSRSKIFDKNSTFGSV (26 MER)
7. FcRH5.1: CTADNGFGPQRSEVVSLFVTVPVSRPILTLRVPRAQAV (38 MER)
8. FcRH5.2: AQHSDTISLSVIVPVSRPILTFRAPRAQA (29 MER)
9. MCL1.1: GAEVPDVTATPARLLFFAPTRRAAPLEEM (29 MER)
10. RHAMM 2: RLNAALREKTSLSANNATLEKQLIELTRTNELLKSK (36 MER)
11. RHAMM 3: SENGNQKNLRILSLELMKLRNKRETKMRGMMAKQEGME (38 MER)
12. RHAMM 4: AHTQATLLLQEKYDSMVQSLEDVTAQFESYKA (32 MER)
13. SLAMF7.2: DVIYTWKALGQAANESHNGSILPISWRWGESDMTFICVAR (40 MER)
14. SLAMF7.5: LGLFLWFLKRERQEENNPKGRSSKYGLL (28 MER)
15. SOX2.1: NSPDRVKRPMNAFMVWSRGQRRKMAQENPKMHNSEISK (38 MER)
16. SOX2.2: EWKLLSETEKRPFIDEAKRLRALHMKEHPDYKYRPRRK (38 MER)
17. XBP(S)1.1: AAAPNPADGTPKVLLLSGQPASAAGAPAGQ (30 MER)
18. XBP(S)1.2: SDILLGILDNLDPVMFFKCPSPEPASLEELPEVYPEGP (38 MER)
19. CT45.1: AVDPETVFKRPRECDSPSYQKRQRMALLARK (31 MER)
20. CT45.4: LEGVQGPTAVRKRFFESIIKEAARCMRRDFVKHL (34 MER)
21. MAGEA3: TFPDLESEFQAALSRKVAELVHFLLLK (27 MER)
22. MAGEA4.1: PDAESLFREALSNKVDELAHFLLRKYRAKEL (31 MER)
23. MAGEA6.1: TFPDLESEFQAALSRKVAKLVHFLLLK (27 MER)
24. MAGEA6.3: FVQENYLEYRQVPGSDPACYEFLWGPRALIETSYVKVLHHM (41 MER)
25. MAGEC1.4: SPLQRPVSSFFSYTLASLLQSSHESPQS (28 MER)
26. MAGEC1.5: SSTSSSLSKSSPESPLQSPVISFSS (25 MER)
27. MAGEC1.8: NSSPPRYEFLWGPRAHSEVIKRKVVEFLAMLKNTVPI (37 MER)
28. NY-ESO-1.1: ARGPESRLLEFYLAMPFATPMEAELARRSLAQDAPPL (37 MER)
29. NY-ESO-1.2: VPGVLLKEFTVSGNILTIRLTAADHRQL (28 MER)
30. WT1.1: RSASETSEKRPFMCAYPGCNKRYFKLSHLQMH (32 MER)

7

Supplementary Figure 1: Synthetic peptides were designed for different antigens based on predictive algorithms. (**A**) An example depicting the methodology used to design peptides. Immunogenic heat map that recognizes regions with high binding affinity for MHC I (blue letters) and MHC II (area highlighted in red) grooves for antigen, CT45 (brackets indicate the designed peptide sequence). (**B**) The peptide sequences for different antigens (17–41 mers) were synthesized that consist of overlapping regions for MHC I and MHC II binding. The peptides: 1–18 are designed from antigens that are overexpressed in MM; peptides 19 through 30 were constructed from cancer testis antigens. The list consists of antigens (MUC1 (SEA1,2,3), CD38, FcRH5, RHAMM, SLAMF7, SOX2, XBP(S)1, CT45, MAGEA6, MAGEC1 and NY-ESO-1) that showed more than one region with overlapping MHC I & II hotspots that could be synthesized. To indicate that the peptides are synthesized from the same antigen, the peptides are labelled accordingly (e.g., CD38.1, CD38.2).

MUC1 Cocktail	Cocktail 1	Cocktail 3	Cocktail 4
	SEA 1	SLAMF7.2	RHAMM 2
SEA1	MAGEA6.1	SLAMF7.5	RHAMM 3
SEA2	MAGEA6.3	FcRH5.1	RHAMM 4
SEA3	CT45.4	FcRH5.2	NYESO 1.1
	SEPT 9.1	BCMA2	NYESO 1.2

Supplementary Figure 2: Four peptide cocktails were used for subsequent experiments. Based on the data obtained following treatment of healthy donor PBMCs with single peptides, four different peptide cocktails were designed to assess the ability of different antigens to co-operatively induce T cell responses from PBMCs isolated from healthy donors or MM patients.

A

Cocktail 1

HD	1		2		3		4		5	
	CD4	CD8	CD4	CD8	CD4	CD8	CD4	CD8	CD4	CD8
T_{EM}	49	53	52	68	57	50	67	48	100	96
T_{CM}	47	42	43	15	41	49	33	49	0	0

MM	1		2		3		4		5	
	CD4	CD8	CD4	CD8	CD4	CD8	CD4	CD8	CD4	CD8
T_{EM}	77	69	76	76	96	94	60	42	100	97
T_{CM}	22	30	23	19	4	3	21	57	0	1

B

Cocktail 3

HD	1		2		3		4		5	
	CD4	CD8	CD4	CD8	CD4	CD8	CD4	CD8	CD4	CD8
T_{EM}	31	64	54	64	56	54	79	64	Insufficient Cells	
T_{CM}	60	26	36	10	41	44	20	34	Insufficient Cells	

MM	1		2		3		4		5	
	CD4	CD8	CD4	CD8	CD4	CD8	CD4	CD8	CD4	CD8
T_{EM}	68	79	90	76	93	89	Insufficient Cells		100	94
T_{CM}	30	19	9	18	7	7	Insufficient Cells		0	1

C

Cocktail 4

HD	1		2		3		4		5	
	CD4	CD8	CD4	CD8	CD4	CD8	CD4	CD8	CD4	CD8
T_{EM}	42	52	57	72	61	24	74	45	37	47
T_{CM}	51	42	33	12	37	75	25	51	0	0

MM	1		2		3		4		5	
	CD4	CD8	CD4	CD8	CD4	CD8	CD4	CD8	CD4	CD8
T_{EM}	78	57	83	70	92	84	Insufficient Cells		100	96
T_{CM}	22	42	16	23	7	13	Insufficient Cells		0	1

Supplementary Figure 3: Culture activation generates T_{EM} and T_{CM} populations in both $CD4^+$ and $CD8^+$ T cells. Table depicting the percentage of T_{EM} and T_{CM} for $CD4^+$ and $CD8^+$ T cells obtained at the end of the culture period following treatment with cocktails (**A**) CT1, (**B**) CT3 and (**C**) CT4.

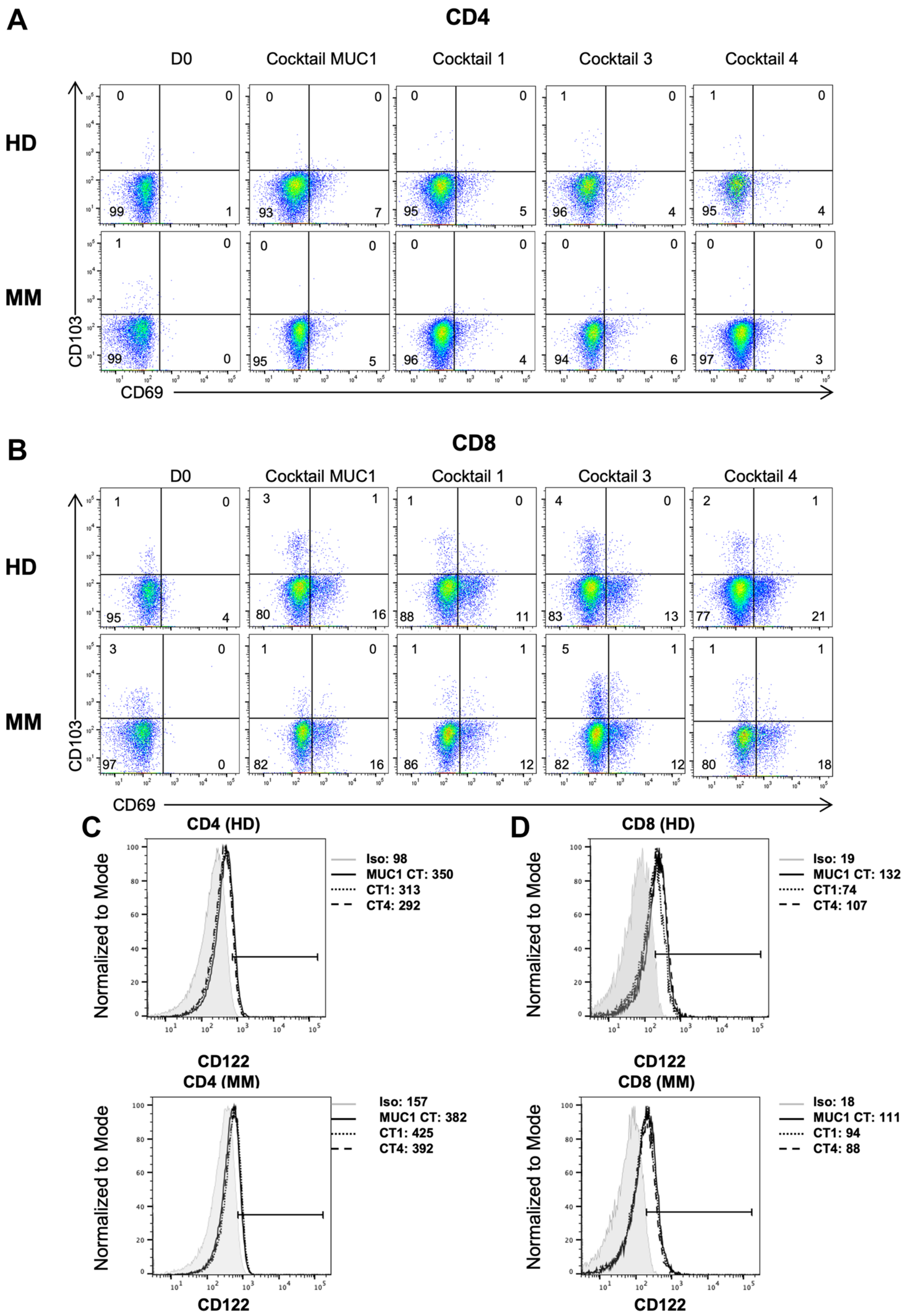

Supplementary Figure 4: Stimulation with peptide cocktail leads to enhanced expression of T_{RM} markers, CD69 and CD103, on $CD4^+$ and $CD8^+$ T cells. (**A**) Expression of CD69 and CD103 on $CD4^+$ T cells or (**B**) $CD8^+$ T cells on D0 or on D19 following stimulation of PBMCs isolated from healthy donor (HD) or MM patient (MM) with either MUC1 Cocktail, Cocktail 1, Cocktail 3 or Cocktail 4. Representative data are shown. CD122 expression on $CD4^+$ T cells (**C**) and $CD8^+$ T cells (**D**) were generated following exposure of PBMCs from HD (top panel) and MM patient (bottom panel) to MUC1 cocktail (solid line histogram), Cocktail 1 (dotted line histogram), and Cocktail 4 (dashed line histogram). The isotype control is depicted by grey histogram. Data representative of four individuals (2 HDs, 2 MM patients).

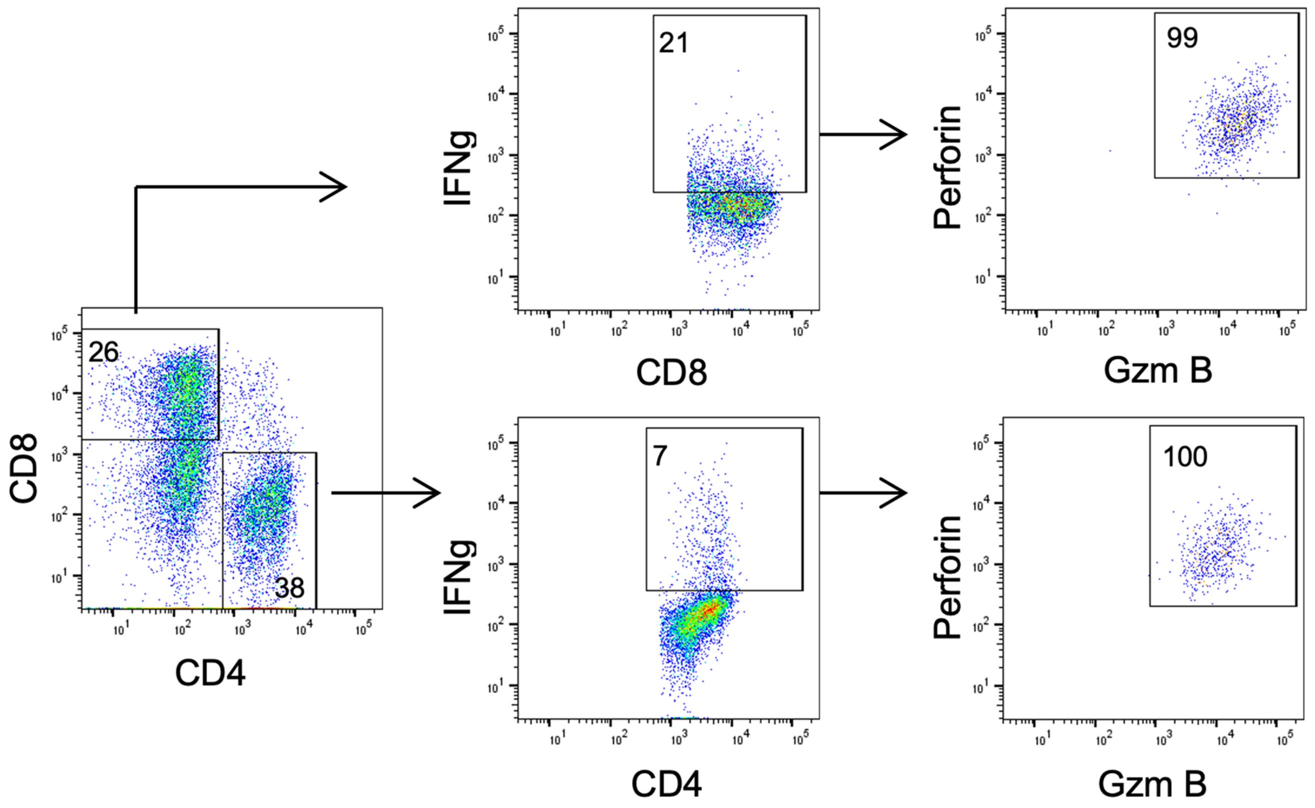

Supplementary Figure 5: Effector memory (T_{EM}) and central memory (T_{CM}) $CD4^+$ and $CD8^+$ T cells possess anti-tumor profile. Representative dot plot showing the gating hierarchy to define different functional subsets of $CD4^+$ and $CD8^+$ T cells. First, viable cells were gated based on the absence of UV Blue stain. These cells are then gated on CD3 and then on CD8, which was used to define $CD8^+IFN\text{-}\gamma^+$. The expression of perforin and granzyme B was examined on $CD8^+IFN\text{-}\gamma^+$. Similar strategy was used for $CD4^+$ T cells. Representative data are shown.
